# A Good Fire Insurance Co.

**Published:** 1876-06

**Authors:** 


We publish the annual statement of the AMERICAN INSURANCE COMPANY OF CHICAGO, a company specializing the insurance of dwelling houses and farm property. Through good management and honorable dealing this company has become one of the most reliable and popular in the country. It has gross assets amounting to nearly three million dollars, and a surplus over capital of over $700,000. It was the only local company that survived the Chicago fire, and its officers and directors are among the most honorable and responsible citizens of that city. Companies that restrict their business to this class of risks are by far the safest, since the loss by any conflagration is necessarily limited. We cheerfully recommed this company to our readers throughout the country, and particularly to those residing in the Western States.

In this connection it is not out of place for us to refer to an attempt which has recently been made to injure the credit of the "American." It seems that by some singular arrangement, Mr. Row, Insurance Commissioner of Michigan, was induced to revoke the license of the Company to transact business in that State; whereupon the American at once invited the Insurance Commissioners of Ohio, Missouri, Illinois, and Iowa to make an examination of its affairs, which was done, resulting in the entire vindication of the Company. After having thus shown the entire falsity of the charges against it, and having concluded to apply for re-admission to the State of Michigan, the Company invited Mr. Row to make a thorough and rigid examination of its condition, the result of which is explained in the following letter from the Secretary of the Company:

"OFFICE OF THE AMERICAN INSURANCE COMPANY,<br>CHICAGO, April 26, 1876.

"HON. SAMUEL H. ROW, *Commissioner of Insurance for the State of Michigan:*

"DEAR SIR—On the fourteenth day of February last you very hastily revoked the authority under which this Company had been doing business in the State of Michigan for six years past. Immediately following the publication of your action, the Insurance Departments of other States in which this Company had been doing business instituted, by competent and able experts, patient and thorough examination into the affairs of this Company, and, without exception, favorable reports were made, and the solvency of the Company fully indorsed by every State in which the Company was doing business, except the State of Michigan. After such favorable reports from all the other States, we then sent our 'statement' to you with a request that you make a personal and thorough examination of the condition of this Company. Upon such request you have this day arrived in Chicago, and announced yourself as ready to proceed in the examination, but have, as a condition precedent, required the Company to place at your disposal, and subject to your order, the sum of eight hundred dollars ($800.00) for such disbursements and expenses as you may feel disposed to make in and about the prosecution of such investigation. Your request has been laid before the directors of this Company, and without any dissent they utterly decline and refuse to accede to your demand, and respectfully notify you that the application to you for a renewal of license to do new business in the State of Michigan by the American Insurance Company is hereby withdrawn.

"Yours respectfully, CHAS. L. CURRIER, Sec'y."

That a public officer should make such a bold demand, and throw himself so completely open to the suspicion which naturally suggests itself to every fair-minded man, and that the Company did right in its refusal to accede to such a peculiar proposition, will be probably allowed by all, and while State Supervision of Insurance as at present administered in Michigan has received a pretty thorough exposition, the old American Insurance Company, of Chicago, stands better to-day, in the estimation of the public, than ever before.

